# Five-year outcomes after IVIG for mild cognitive impairment due to alzheimer disease

**DOI:** 10.1186/s12868-021-00651-2

**Published:** 2021-08-06

**Authors:** Shawn Kile, William Au, Carol Parise, Kimberley Rose, Tammy Donnel, Andrea Hankins, Yvonne Au, Matthew Chan, Azad Ghassemi

**Affiliations:** 1 Sutter Neuroscience Institute, 2800 L Street, Suite 500, Sacramento, CA 95816 USA; 2 Sutter Institute for Medical Research (SIMR), Sacramento, CA USA; 3 Sutter Imaging, Neuroradiology, Sacramento USA

**Keywords:** Intravenous immunoglobulin (IVIG), Mild cognitive impairment (MCI), Alzheimer’s disease, Ventricular volume

## Abstract

**Background:**

The purpose of this study was to assess the five-year treatment effects of a short course of intravenous immunoglobulin (IVIG) in subjects with mild cognitive impairment (MCI) due to Alzheimer disease (AD).

**Methods:**

Fifty subjects 50 to 84 years of age with MCI due to AD were administered 0.4 g/kg 10% IVIG or 0.9% saline every two weeks x five doses in a randomized double-blinded design as part of a two-year study. Twenty-seven subjects completed an additional three-year extension study. MRI brain imaging, cognitive testing, and conversion to dementia were assessed annually. Participants were stratified into early MCI (E-MCI) and late MCI (L-MCI). The primary endpoint was brain atrophy measured as annualized percent change in ventricular volume (APCV) annually for five years. ANOVA was used to compare annualized percent change in ventricular volume from baseline between the groups adjusting for MCI status (E-MCI, L-MCI).

**Results:**

Differences in brain atrophy between the groups, which were statistically significant after one year, were no longer significant after five years. IVIG-treated L-MCI subjects did demonstrate a delay in conversion to dementia of 21.4 weeks.

**Conclusion:**

An eight-week course of IVIG totaling 2 g/kg in MCI is safe but is not sufficient to sustain an initial reduction in brain atrophy or a temporary delay in conversion to dementia at five years. Other dosing strategies of IVIG in the early stages of AD should be investigated to assess more sustainable disease-modifying effects.

*Trial registration* ClinicalTrials.gov NCT01300728. Registered 23 February 2011.

## Background

Alzheimer disease (AD) is a neurodegenerative disorder which causes dementia and brain atrophy associated with the accumulation of pathological Aβ and tau proteins. Intravenous immunoglobulin (IVIG) contains naturally occurring polyclonal antibodies to both monomeric and oligomeric Aβ and has been investigated as a potential disease-modifying strategy for AD [1–6]. IVIG has other potentially important mechanisms of action for AD including also containing tau antibodies and having regulatory effects on microglia [1, 7].

Animal models have demonstrated amyloid clearance from the brain with IVIG when analyzed with ex vivo and in vivo assays [8–10]. An exploratory substudy showed a reduction in amyloid using Florbetapir PET after 18 months of IVIG treatment 0.4 g/kg every two weeks [11]. In addition, a proof of mechanism study demonstrated that an eight-week course of 0.4 g/kg IVIG every two weeks resulted in measurable removal of amyloid from the brain and retina in subjects with mild cognitive impairment (MCI) due to AD [12].

While open label and Phase I studies [13, 14] of IVIG for AD were promising, Phase II and III studies of IVIG failed to reach significant primary endpoints in mild to moderate AD dementia [11, 15]. We conducted the first study investigating administration of IVIG in the earliest clinical stage of AD, MCI. The details of this randomized, double-blinded, placebo-controlled trial have been previously published [16]. Subjects with MCI due to AD were randomized to receive either 0.4 g/kg of IVIG or 0.9% saline solution every 14 days x five infusions. We chose the infusion interval based upon the 9.8-day serum half-life of Aβ antibodies in IVIG, as well as prior studies having demonstrated that IVIG 0.4 g/kg every two weeks may be the most optimal dosing for AD [15, 17]. Due to the limited availability and cost of IVIG, we decided to test the shortest reasonable course. Our results showed that there was less brain atrophy in participants given IVIG when compared with placebo after one year when adjusted for baseline cognition; however, this difference was not statistically significant at two years. We also found less cognitive decline and conversions to dementia at one year in late MCI (L-MCI) subjects treated with IVIG. Subjects were offered the opportunity to continue in an extension study for another three years in order to assess five-year outcomes.

In a relevant five-year retrospective study of patients previously treated with even one course of IVIG for various indications, Fillet and colleagues demonstrated a 42% reduction in the risk of developing AD dementia [18]. The purpose of this study was to assess for similar long-term effects of IVIG given to subjects with MCI.

## Methods

### Study design and participants

This was a single-center, 1:1 randomized, double-blind, placebo-controlled outpatient extension trial where subjects with MCI due to AD were randomized to receive either 0.4 g/kg of IVIG or 0.9% saline solution every 14 days x five infusions [16]. Subjects were patients 50 to 84 years of age referred to a community-based neurology clinic, which included a specialized memory clinic. Subjects were diagnosed with single or multi-domain amnestic MCI according to the Petersen criteria [19] and the National Institute of Neurological and Communicative Disorders—Alzheimer’s Disease and Related Disorders Association (NINCDS-ADRDA) and National Institute on Aging—Alzheimer’s Association (NIA-AA) criteria for MCI due to AD [20]. All screening visit MRIs were reviewed by the primary investigator to ensure brain atrophy patterns, such as mild hippocampal atrophy, were consistent with the diagnosis of MCI due to AD. Participants had the option of undergoing a lumbar puncture for evaluation of CSF Aβ_42_, tau, or p-tau to assess underlying pathology.

Other inclusion criteria were Clinical Dementia Rating (CDR) global score of ≤ 0.5, Mini-Mental State Examination, 2nd edition (MMSE) of 24 to 30 using the higher score with either serial 7 s or WORLD backwards for inclusion purposes. MMSE score was then tracked throughout the study using only serial 7 s. Medication dosages for non-excluded medical conditions must have been stable for at least 30 days prior to screening. Concurrent treatment with cholinesterase inhibitors or memantine was an exclusion, however discontinuation of any of these agents 90 days prior to screening was permitted. Subjects were permitted to start a cholinesterase inhibitor and/or memantine if conversion to dementia occurred during the study. A family member or friend was required to participate as a collaborative informant for the duration of the study. Subjects were excluded if there were MRI brain abnormalities such as multiple microhemorrhages, infarcts, or moderate to severe cortical atrophy. Other exclusion criteria included conditions that would make it unsafe to administer IVIG, such as IgA deficiency. Participants who completed two years of the study were given the option to enroll for an additional three years.

#### Endpoints

The primary endpoint was brain atrophy measured as annualized percent change in ventricular volume (APCV) annually for five years. Ventricular volume was measured using NeuroQuant 2.3, which is the updated version of software used in our previously published paper [16].

Secondary endpoints included change in cognitive performance annually as measured by ADAS-Cog, MMSE, and CDR-SB.

Exploratory analysis included subdividing subjects into early MCI (E-MCI) and late MCI (L-MCI) groups using the baseline CDR-SB. E-MCI was defined as a CDR-SB of 1.0 or less to reflect the earliest stages of decline and commensurate with mean CDR-SB scores found in other studies for E-MCI and L-MCI participants [4–6]. Conversion from L-MCI to AD was also examined and based on NINCDS-ADRDA and NIA-AA criteria for the diagnosis of AD [3] and supported by CDR global score of ≥ 1.0.

The study was approved by the Sutter Health Institutional Review Board.

### Procedures

The details of the study procedures have been previously published [16]. Ventricular volume was measured annually for five years. MMSE, CDR, and ADAS-Cog, along with evaluation for conversion to AD, were conducted at 4-month intervals for 2 years and annually thereafter.

### Statistical analysis

Descriptive statistics, histograms, Q-Q, and scatter plots were used to assess if the characteristics of the data were suitable for parametric statistics. Analysis of variance (ANOVA) and the χ2 Test of Independence were used to compare baseline characteristics between the groups.

#### Primary and secondary endpoints

ANOVA was used to compare annualized percent change in ventricular volume from baseline between the groups adjusting for MCI status (E-MCI, L-MCI). Linear mixed models with all available cases was used to assess the secondary endpoints. The fixed effects of baseline age (centered at the mean), baseline CDR-SB, time (included as a continuous variable), group (IVIG or placebo), and the time X group interaction were included in the models.

#### Exploratory endpoints

Statistical testing was not conducted for comparisons that were stratified by E-MCI and L-MCI due to insufficient cases in some strata. Kaplan–Meier survival analysis was used to compare conversion to AD between the groups of subjects classified with L-MCI. Statistical analyses were conducted using IBM SPSS Version 21.0 [21].

## Results

**Fig 1.**
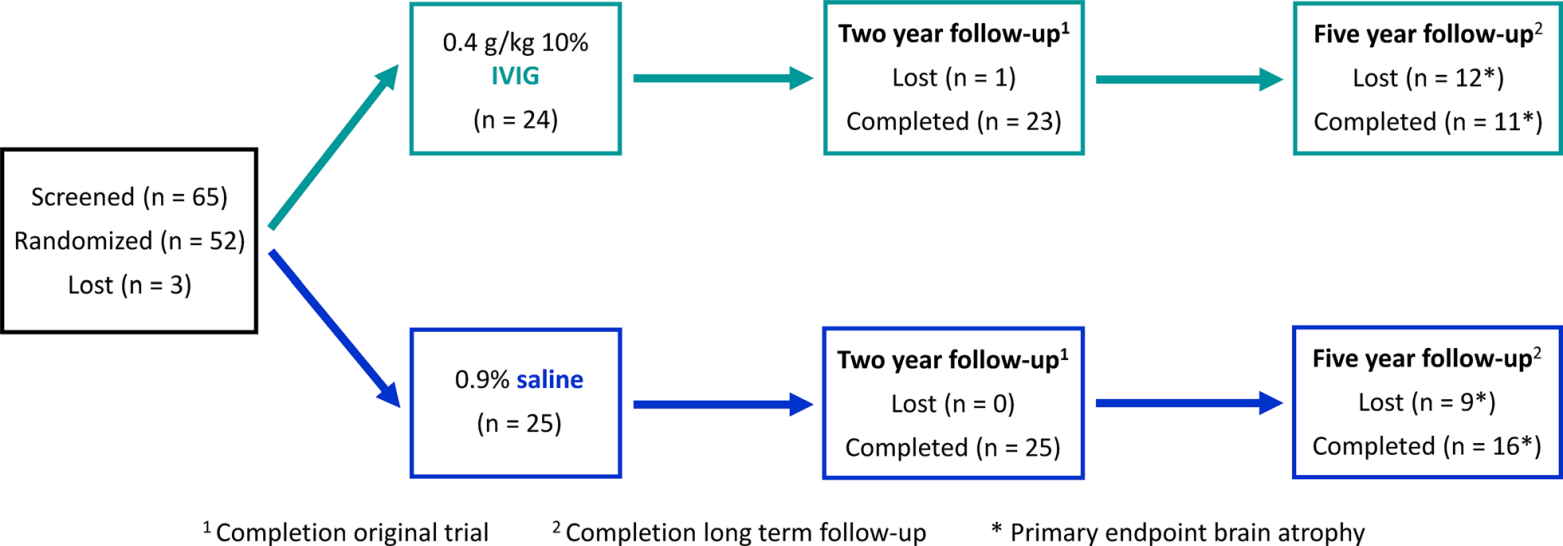
Consort flow diagram

Sixty-five subjects were screened from 2011 to 2013; 52 subjects qualified for randomization and 50 completed infusions of IVIG or placebo. One subject was subsequently diagnosed with primary progressive aphasia and was removed from the study leaving 49 subjects to complete the initial trial. Forty of the 49 subjects elected to continue for the additional three years. Figure 1 shows the flow of subjects through the trial.

After five years, 30 subjects completed the ADAS-Cog and the CDR-SB, 32 completed the MMSE, and 27 subjects completed MRI for volumetric measures. The reasons that subjects who elected to continue but did not complete the study included death (n = 2; 4.9%), subject/caregiver decision (n = 4; 9.8%), and investigator decision (n = 2; 4.9%).

Thirty-four subjects (17 IVIG; 17 placebo) opted for the lumbar puncture at baseline; there were no significant differences between the groups in CSF Aβ_42_, tau, or p-tau. Eighteen subjects with these measures completed five years of the trial.

Table 1 displays the baseline demographic characteristics of subjects who were enrolled in the study and received at least one infusion of IVIG or placebo. There were no statistically significant differences (p > 0.05) in any of the baseline characteristics between the groups.

**Table 1:** Demographic and clinical characteristics of randomized subjects at baseline

	IVIG n = 24 (49.0%)		Placebo n = 25 (51.0%)	
	n (%)	Mean ± SD	n (%)	Mean ± SD
Age		72.20 ± 7.91		72.34 ± 7.36
Female	14 (58.3)		14 (56.0)	
Education				
< 12 years	0		0	
12 years	2 (9.5)		3 (13.6)	
> 12 to 16 years	9 (42.9)		11 (50.0)	
> 16 years	10 (47.6)		8 (36.4)	
BMI		25.48 ± 5.44		26.96 ± 4.26
Comorbids				
Asthma/COPD	4 (16.7)		2 (8.0)	
Diabetes	5 (20.8)		5 (20.0)	
Hypertension	10 (41.7)		8 (32.0)	
Hyperlipidemia	13 (54.2)		10 (40.0)	
CAD/PVD	4 (16.7)		6 (24.0)	
*APOE ε*4	15 (62.5)		14 (56.0)	
Aβ_42_ < 482 pg/mL^a^	16 (94.1)		15 (88.2)	
Aβ_42_ pg/mL^a^		294.00 ± 96.55		353.41 ± 112.99
tau pg/mL^a^		107.47 ± 58.65		96.18 ± 65.47
p-tau pg/mL^a^		43.59 ± 19.90		39.47 ± 30.27
Ventricular volume (cm^3^)		53.64 ± 26.84		51.72 ± 22.28
MMSE		26.75 ± 2.15		26.44 ± 2.60
ADAS-cog		10.21 ± 4.38		10.28 ± 5.67
CDR-SB		1.96 ± 0.95		1.60 ± 0.90
MCI Stage				
Early (CDR-SB ≤ 1.0)	6 (25.0)		13 (52.0)	
Late (CDR-SB > 1.0)	18 (75.0)		12 (48.0)	

*ADAS-Cog* Alzheimer’s Disease Assessment Scale-cognitive subscale, *Aβ* β-amyloid, *BMI* body mass index, *CAD* coronary artery disease, *CDR-SB* Clinical Dementia Rating-Sum of Boxes, *COPD* chronic obstructive pulmonary disease, *IVIG* intravenous immunoglobulin, *MCI* mild cognitive impairment, *MMSE* Mini-Mental State Examination, *PVD* peripheral vascular disease

^a^CSF collected from 34 participants (17 IVIG; 17 placebo) who opted to participate with lumbar puncture

### Adverse events

There were no drug-related adverse events and no amyloid-related imaging abnormalities such as hemorrhage or vasogenic edema.

### Brain atrophy

**Fig. 2:**
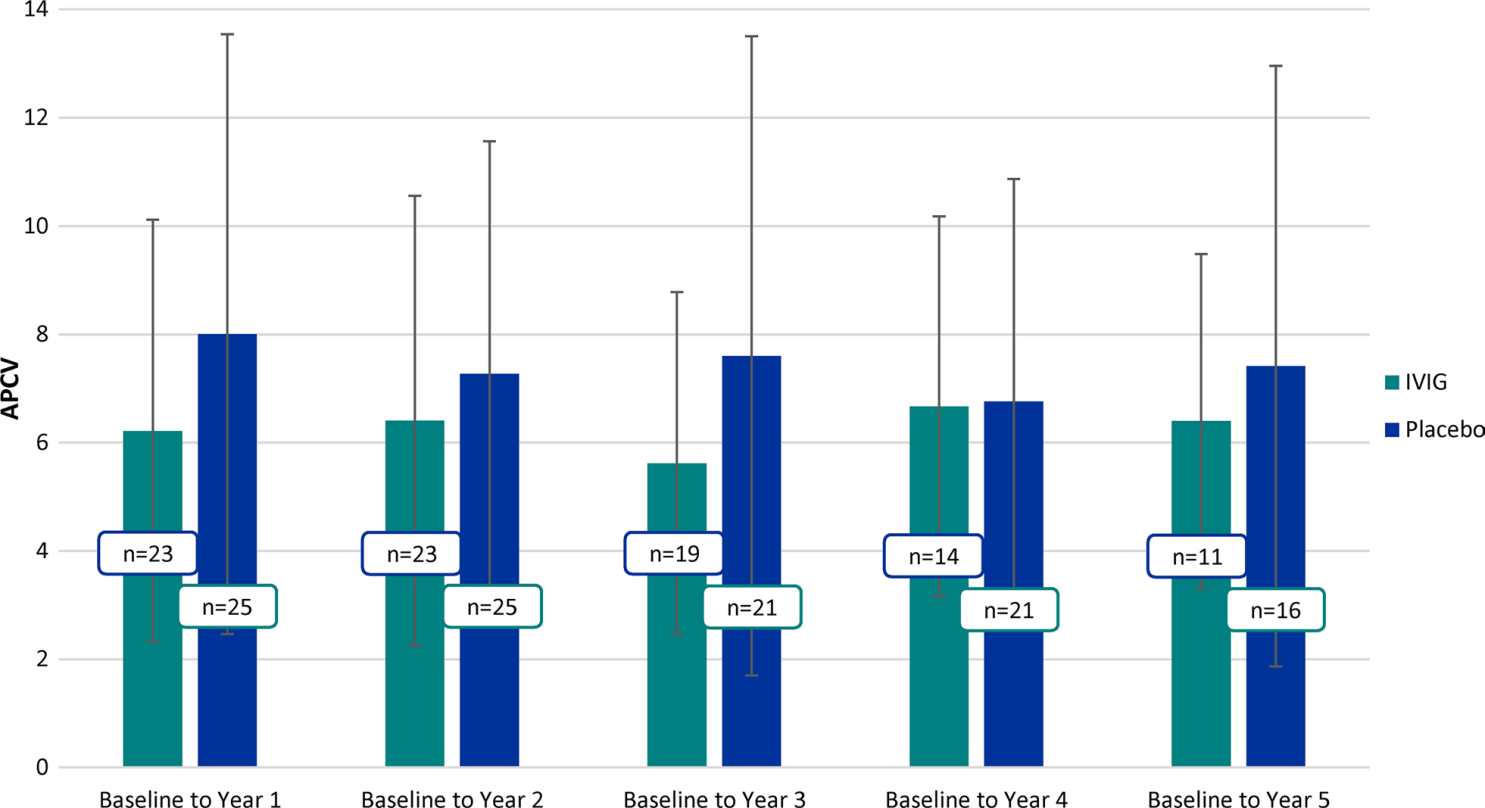
Annualized percent change in ventricular volume (cm^3^) at 1, 2, 3, 4, and 5 years following IVIG or placebo

**Fig. 3:**
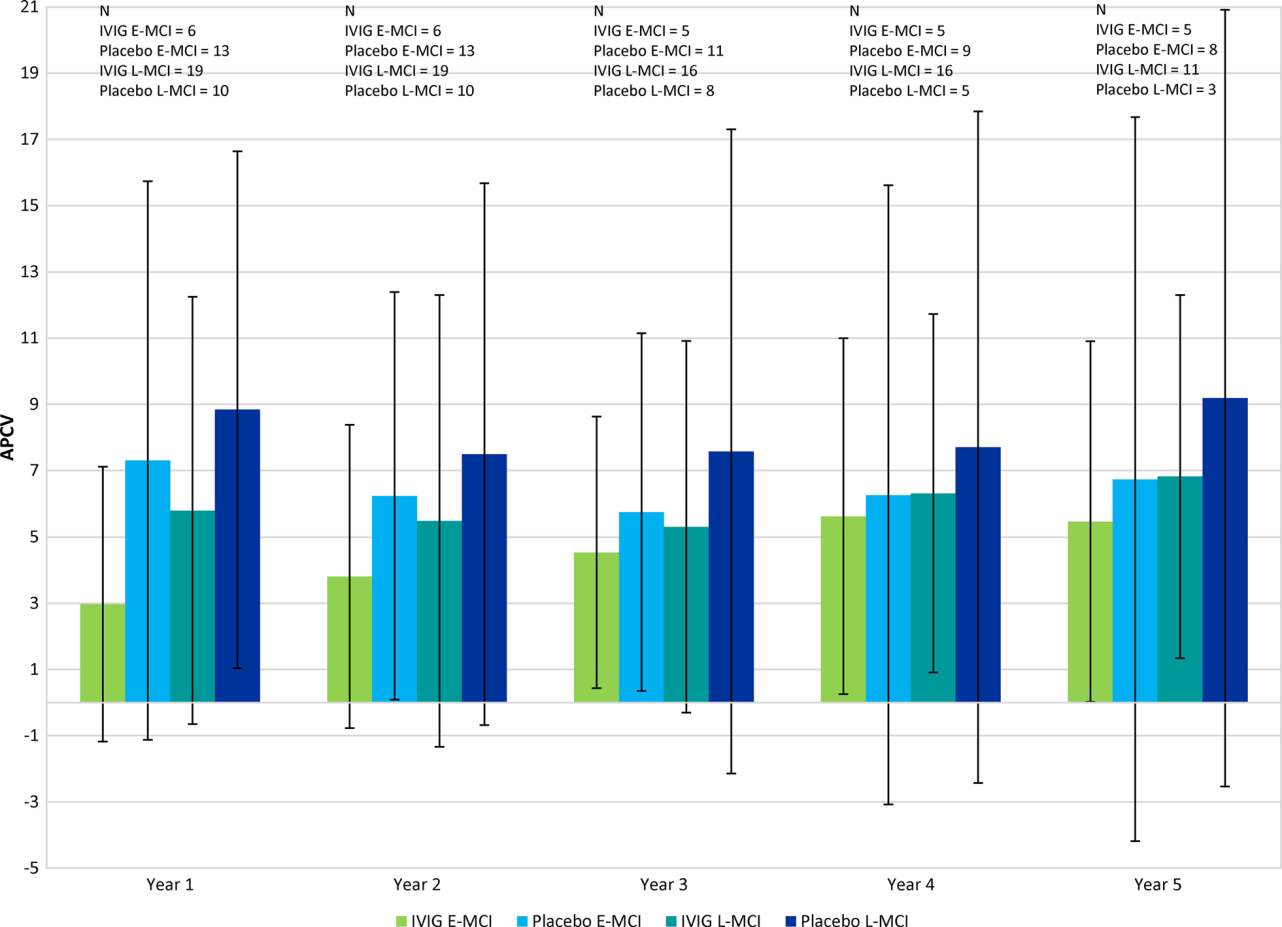
Annualized percent change in ventricular volume (cm^3^) at 1, 2, 3, 4, and 5 years following IVIG or placebo stratified into E-MCI and L-MCI mild cognitive impairment

Figure 2 shows ventricular APCV between baseline and 5 years. IVIG showed lower brain atrophy in all years but none of the differences were statistically significant (p > 0.05). Figure 3 shows the APCV between the groups stratified by E-MCI and L-MCI. The effects of IVIG were most pronounced in L-MCI. It should be noted that due to the update in NeuroQuant to version 2.3, the exact values for baseline, year 1 and year 2 volume varied when compared to our previous publication.

### Cognition

**Fig. 4:**
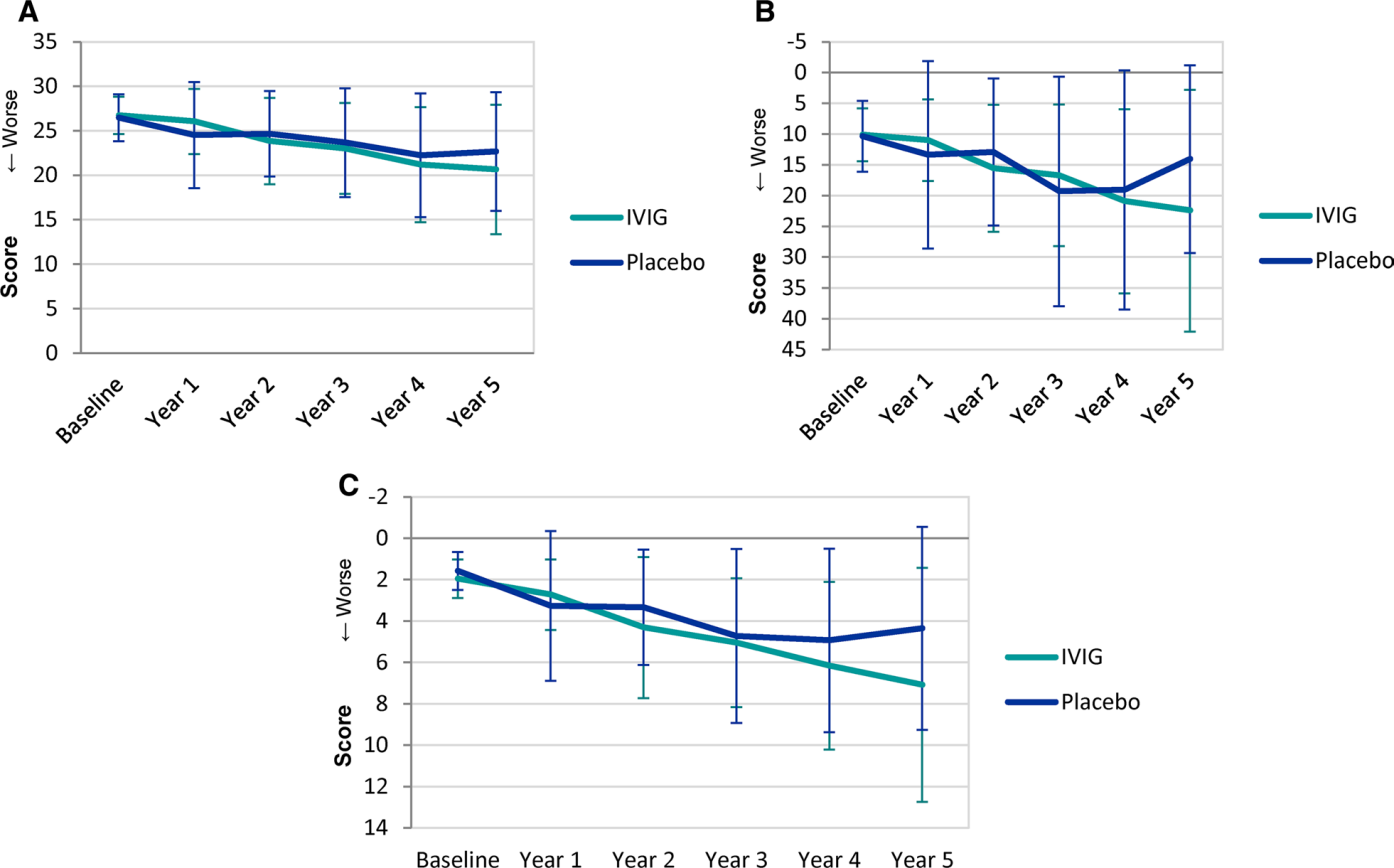
MMSE (Panel A), ADAS-Cog (Panel B), and CDR-SB (Panel C) at baseline and 1, 2, 3, 4, and 5 years following IVIG or placebo

Figure 4(A–C) shows mean MMSE (Panel A), ADAS-Cog (Panel B), and CDR-SB (Panel C) for each group over the six time points. The figure contains the 11 cases in the placebo group and the 16 cases in the IVIG group that had data for all time points. There were no statistically significant differences between the groups on any of the cognitive measures.

**Fig. 5:**
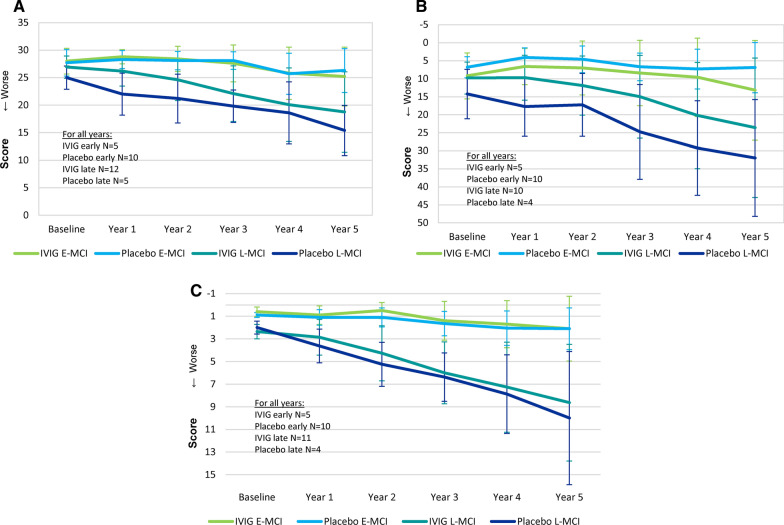
MMSE (Panel A), ADAS-Cog (Panel B), and CDR-SB (Panel C) at baseline and 1, 2, 3, 4, and 5 years following IVIG or placebo stratified into E-MCI and L-MCI

Figure 5(A–C) shows the mean MMSE (Panel A), ADAS-Cog (Panel B), and CDR-SB (Panel C) scores for both groups after five years of follow up stratified by MCI status. In year five, the placebo group included 10 subjects classified as E-MCI and 4 classified as L-MCI. For the IVIG group, 5 subjects were E-MCI and 11 were L-MCI. The subgroups demonstrate more favorable cognitive scores in L-MCI subjects that received IVIG than subjects in the placebo group on the ADAS-cog, CDR-SB, and MMSE at five years. L-MCI placebo subjects had an average ADAS-cog total score of 32.00 at five years, compared to the IVIG average of 23.60. L-MCI placebo subjects had an average MMSE total score of 15.40 at five years, compared to the IVIG average of 18.75. L-MCI placebo subjects had an average CDR-SB score of 10.00 at five years (3.63 at one year), compared to the IVIG average of 8.64 at five years (2.86 at one year).

### Conversion to AD dementia

**Fig. 6:**
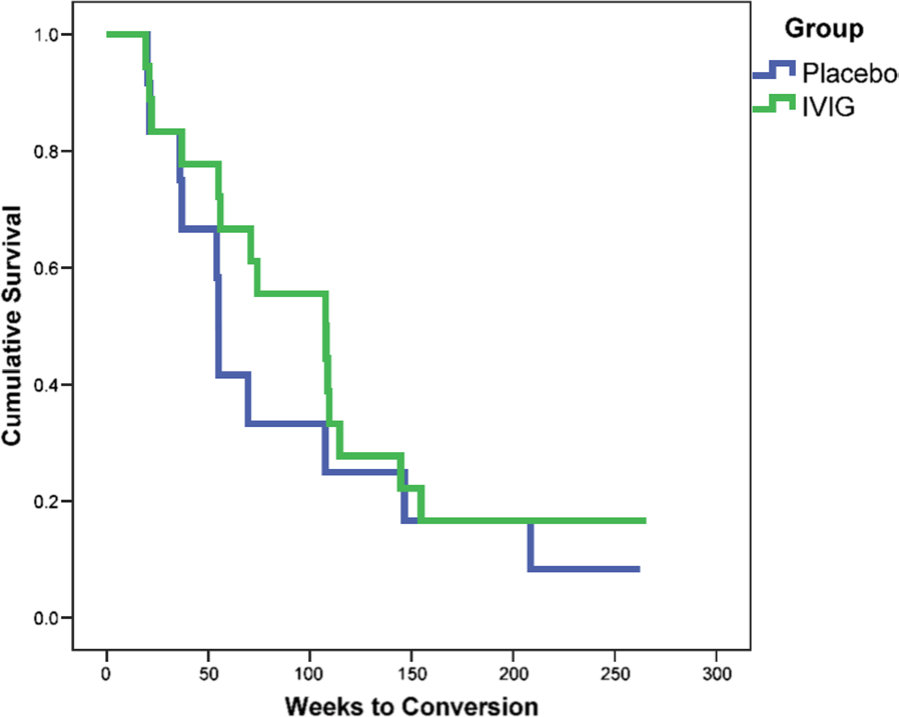
Kaplan–Meier survival analysis for conversion to Alzheimer’s disease dementia for subjects with L-MCI following IVIG or placebo

After five years, 11 of 12 (92%) L-MCI subjects in the placebo group and 15 of 18 (83%) subjects in the IVIG group converted to AD. The IVIG group took 110.8 weeks to convert from L-MCI to dementia compared with 89.4 weeks in the placebo group (Fig. 6) which amounts to a 21.4 week delay in time to conversion from L-MCI to dementia.

## Discussion

The results of this study indicate that an eight-week course of IVIG totaling 2 g/kg does not significantly reduce brain atrophy or prevent conversion to dementia after five years. The results published after two-years showed that this single course of IVIG for MCI significantly reduced brain atrophy at one year [16]. This trend was still present after five years, but there were no statistically significant differences between the groups in either brain atrophy or cognition. This study has other positive trends that are worth highlighting.

### IVIG is safe

Consistent with previous investigations, IVIG appears to be safe with no major adverse events or amyloid related imaging abnormalities [2, 15, 16]. This is a noteworthy contrast to the monoclonal antibody treatments for AD, including recently FDA approved aducanumab, in which amyloid related imaging abnormalities of cerebral edema and hemorrhages have been consistently observed. [22–24].

### IVIG shows a trend in delay of conversion to AD dementia

After five years, a smaller proportion of subjects with L-MCI treated with IVIG converted to AD dementia compared to placebo. This temporary delay is noteworthy as even a modest delay in conversion to dementia can have a significant impact on the growing prevalence of AD. Our five-year results did not match the results of the retrospective study by Fillit and colleagues which showed a 42% reduction in AD dementia in subjects who had IVIG treatment for any indication in the preceding five years [18]. This may be due to the mean number of IVIG infusions in the Fillit study was 14, compared to only five infusions in our trial.

### IVIG shows a trend in reduction of cognitive decline in subjects with L-MCI

IVIG treated L-MCI subjects had less decline on all cognitive measures (Fig. 5). IVIG demonstrated a 69.3% reduction of decline in L-MCI subjects over placebo at 12 months, and a 21.5% reduction of decline over placebo at five years. For comparison, aducanumab demonstrated a 22% reduction of decline on the CDR-SB when compared with placebo at 18 months [22]. The L-MCI subgroup in the present investigation is most comparable to the aducanumab Phase III studies which included patients with MCI and mild dementia. The CDR-SB scores for L-MCI (Fig. 5C) can be used for comparison to the cognitive results for aducanumab [22]. Because the present investigation yielded no statistically significant differences in the overall results, formal statistical analysis within the L-MCI and E-MCI subgroups were not warranted.

This study has limitations. Amyloid PET imaging was not used as a biomarker of AD as it was not readily available when the study originally enrolled, however our selection of subjects with MCI specifically due to AD was validated by CSF with 92.9% of tested participants demonstrating an Aβ pattern consistent with AD. In addition, because this was originally designed as a two-year study, it did not have the sample size needed to account for the anticipated attrition of a five-year study, which limits the statistical power. The additional three years of data provide valuable point estimates which can be used for the design of larger trials.

## Conclusion

This study demonstrated that a short course of IVIG for MCI does not significantly reduce brain atrophy or prevent conversion to dementia after five years. Additional doses of IVIG are likely needed to maintain the significant but finite treatment effects described in our initial two-year study. Future studies should continue to investigate IVIG as a viable and safe treatment option for the early stages of AD.

## Data Availability

The data generated for the current study are not publicly available since participants did not consent to having their data disclosed outside of the institution.

## Abbreviations

AD: Alzheimer disease
ADAS-Cog: Alzheimer’s disease assessment scale-cognitive subscale
CDR-SB: Clinical dementia rating scale-sum of boxes
E-MCI: Early mild cognitive impairment
IVIG: Intravenous immunoglobulin
L-MCI: Late mild cognitive impairment
MCI: Mild cognitive impairment
MMSE: Mini-mental state examination
